# Metropolitan Convalescent Institution

**Published:** 1905-06-03

**Authors:** 


### METROPOLITAN CONVALESCENT INSTITUTION.

### NEW HOME FOR MEN AT BEXHILL.

ON Saturday last, at the invitation of the Board of Management of the Metropolitan Convalescent Institution, a party from London visited Bexhill to inspect the new home for men which has recently been opened. After being entertained at lunch at the Hôtel Métropole, the party were driven in carriages to the new home, which is about three miles to the west of Bexhill.

The building, which is already occupied by patients, stands on an ample site, at a good elevation above the sea-level, and faces southward. When finished it will accommodate 118 patients, but as it now stands room is provided for 71 patients, while the administrative quarters are completed for the full staff. It is hoped that funds will soon be forthcoming to allow the completion of the home.

As planned, the remaining parts, consisting of east and west wings, can be added without interfering with the work of the existing portion.

Opposite the main entrance, which is at the western end of the principal block, is a corridor which traverses the whole length of the building. Opening into the corridor are the following rooms on the right hand, that is to say, occupying the south aspect of the building:—Committee-room, reading and quiet room, general sitting and games room, smoking room. These rooms face on to a verandah, so that patients may have the benefit of the fresh sea air even when the weather is inclement.

On the left of the corridor are a store-room, receiving-room, passage leading to the sanitary pavilion, cloak-room, visitors' room, chaplain's room, medical officer's room, and main staircase with lift. At the eastern end of this corridor, and at right angles with it, is a second corridor, which gives access to the kitchen and servants' block.

The first and second floors of the main block are chiefly devoted to dormitories for the patients, with lavatories and baths in the sanitary block at the rear. The servants' dormitories are placed over the kitchen block.

The building is, as far as practicable, designed to be fire-resisting, and has concrete floors. There are ample subsidiary means of egress provided in case of fire.

The lavatories and baths occupy a pavilion at the rear of the main block, and connected with it by cross-ventilated passages on each floor. A mortuary has been placed near the entrance lodge, at a considerable distance from the main building.

Although three miles from Bexhill, the home has been able to benefit by the town's water and electrical supplies and drainage system. The architects are Messrs. Rowland Plumbe and Harvey, and the building contractors were Messrs. Holloway Brothers.

After seeing the new home a short visit was made to the old home, which is situated on the other side of Bexhill. This home, which has hitherto accommodated both men and women patients, will in future be entirely reserved for the latter, as the new establishment amply provides for the convalescent men.

